# A case of primary aldosteronism with a negative aldosterone-to-renin ratio

**DOI:** 10.1186/s12872-021-02162-8

**Published:** 2021-07-22

**Authors:** Fengyi Liu, Liang Wang, Yanchun Ding

**Affiliations:** 1 Department of Cardiology II, The Second Affiliated Hospital of Dalian Medical University, No.467 Zhongshan Road, Dalian, 116021 Liaoning People’s Republic of China; 2 Department of Urology I, The Second Affiliated Hospital of Dalian Medical University, Dalian, 116021 Liaoning People’s Republic of China

**Keywords:** Primary aldosteronism, Hypertension, Adrenal venous sampling

## Abstract

**Background:**

Primary aldosteronism (PA), as a cause of secondary hypertension, can cause more serious cardiovascular damage than essential hypertension. The aldosterone-to-renin ratio (ARR) is recommended as the most reliable screening method for PA, but ARR screening is often influenced by many factors. PA cannot be easily excluded when negative ARR.

**Case presentation:**

We report the case of a 45-year-old Chinese man with resistant hypertension. Three years ago, he underwent a comprehensive screening for secondary hypertension, including the ARR, and the result was negative. After that, the patient's blood pressure was still poorly controlled with four kinds of antihypertensive drugs, the target organ damage of hypertension progressed, and hypokalaemia was difficult to correct. When the patient was hospitalized again for comprehensive examination, we found that aldosterone levels had significantly increased, although the ARR was negative. An inhibitory test with saline was further carried out, and the results suggested that aldosterone was not inhibited; therefore, PA was diagnosed. We performed a unilateral adenoma resection for this patient, and spironolactone was continued to control blood pressure. After the operation, blood pressure is well controlled, and hypokalaemia is corrected.

**Conclusion:**

When the ARR is negative, PA cannot be easily excluded. Comprehensive analysis and diagnosis should be based on the medication and clinical conditions of patients.

## Background

Primary aldosteronism (PA), as a cause of secondary hypertension, was originally defined and reported by Conn [1]. PA refers to the autonomic secretion of aldosterone in the adrenal cortex, resulting in potassium excretion and increased blood volume in vivo and the inhibition of the activity of the renin angiotensin system. Compared with patients with essential hypertension, the damage to target organs, such as the heart and kidney, in patients with PA is more serious [2, 3]. Therefore, early screening of PA in patients with hypertension is very important.

In PA, a high aldosterone concentration is accompanied by a low level of renin because the negative feedback circuit inhibits the secretion of renin. During the development of PA from the normal renin and aldosterone values of healthy individuals to explicit PA, aldosterone is significantly increased, renin is completely inhibited (which is a continuous process), and the aldosterone-to-renin ratio (ARR) continues to rise [4]. The ARR, first proposed by Hiramatsu et al [5], is recommended as the most reliable screening method for PA. If the ARR is positive, further confirmatory tests should be performed; if it is negative, PA screening can be stopped [6, 7]. ARR screening is often influenced by many factors, such as sex, hypokalaemia, body position, measurement time, salt intake, antihypertensive drugs, renal function and age [4]. In clinical diagnosis, we should combine the ARR and clinical manifestations and laboratory examination results to avoid missed diagnosis of PA. We report a case of PA with a negative ARR.

## Case presentation

A 45-year-old Chinese man had elevated blood pressure for 4 years, and the highest blood pressure was 200/130 mmHg. In 2017, he was hospitalized in our department for adrenal Computed tomography (CT) examination, in which a left adrenal nodule was observed, and ARR screening for PA was negative, with an aldosterone/renin ratio of 0.91 (according to the guideline [6], aldosterone (ng/dl), direct renin concentration (ng/l), and the ARR cut-off point were 3.8, 5.7 and 7.7, respectively) (Table 3). Secondary hypertension was ruled out by laboratory and imaging examinations. The final diagnosis was essential hypertension. He had a history of smoking for 20 years and denied any remarkable family medical history. Many times blood potassium was lower than normal. Nifedipine controlled-release tablets, perindopril and indapamide tablets, metoprolol sustained-release tablets and spironolactone were all used to control blood pressure, but the blood pressure was still poorly controlled, and the antihypertensive drugs were adjusted many times. The patient had occasional dizziness and palpitations, but no chest tightness, no chest pain, no syncope, and no weakness. The patient came to our hospital again due to poor blood pressure control. After 2 weeks of oral administration of nifedipine controlled-release tablets combined with doxazosin, the patient was rehospitalized for further thorough examination.

**Fig. 1:**
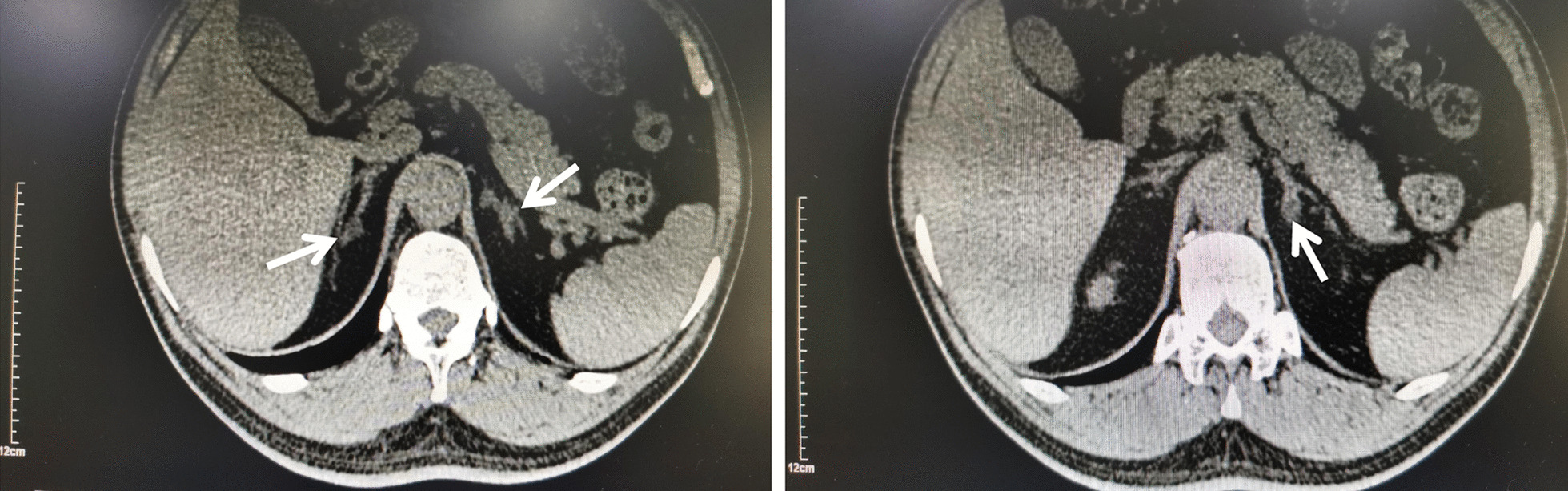
Adrenal computed tomography (CT) scan. The arrows indicate the adenoma

Physical examination results were as follows: pulse, 64 times/min; blood pressure, 165/108 mmHg; body mass index, 25.88 kg/m^2^; heart murmur boundary enlarged to the left; and no other abnormalities. Auxiliary examination results were as follows: the results of routine blood, routine blood coagulation, liver function and thyroid function tests were all normal, as were D-dimer, blood glucose and glycosylated haemoglobin levels. The rhythm of cortisol was normal. The results of the two examinations (in 2017 and 2020) were all abnormal: hypokalaemia, mild renal dysfunction, and positive urine protein (Table 1). In 2017, when the synchronous blood electrolytes were 3.32 mmol/l, the 24-h urine potassium was 32.94 mmol, suggesting increased potassium in urine. In other imaging examinations (Table 2), left ventricular hypertrophy was indicated by cardiac colour Doppler ultrasonography, and the ventricle presented hypertrophic enlargement compared with 2017; carotid artery colour Doppler ultrasonography indicated carotid atherosclerosis, which also indicated the progression of atherosclerosis; renal artery colour Doppler ultrasonography showed no abnormality. CT of the adrenal gland showed that the adrenal glands were both thickened, and multiple round soft-tissue density shadows, such as soft tissue density shadows, could be seen in both adrenal glands. The larger one was located in the left inner branch of the adrenal gland, with a diameter of 8 mm (Fig. 1).

**Table 1:** Biochemical examination results

	Blood potassium	creatinine	Glomerular filtration rate	Urine protein	24-h urinary potassium
2017	3.09 mmol/l	105umol/l	72.5 ml/min	+	32.94 mmol/24 h Synchronous blood potassium:3.32 mmol/l
2020	3.22 mmol/l	117.04umol/l	62.54 ml/min	+

**Table 2:** Auxiliary examination results

	2017	2020
Echocardiography	Left atrium: 48.2 mm Left ventricular end diastolic diameter: 52.3 mm Inter Ventricular septum: 15.4 mm Left ventricular posterior wall thickness: 14.8 mm	Left atrium: 55.3 mm Left ventricular end diastolic diameter: 56 mm Inter Ventricular septum: 12 mm Left ventricular posterior wall thickness: 12.5 mm
Cervical vascular ultrasound	The right carotid artery bifurcation showed mixed plaques, the thickest part was 1.8 mm	The heterogeneous carotid plaque at the thickest part of the right carotid artery is 1.9 mm. Hypoechoic plaques were seen in the posterior wall of the left common carotid artery. The thickest part was 2.5 mm

In 2020, the patient again underwent a postural test (Table 3). Although the ARR was negative (aldosterone/renin was 0.57), the level of aldosterone had increased significantly (> 20 ng/dl), indicating that the inappropriate secretion of aldosterone had increased. Based on this evidence, an inhibitory test with saline (infusion of 2 l of saline within 4 h) was further carried out. As shown in Table 4, aldosterone was more than 10 ng/dl after saline treatment, suggesting that aldosterone was not inhibited. We summarized the clinical features of this patient as follows: hypertension combined with hypokalaemia, poor blood pressure control by various antihypertensive drugs, significant progress of target organ damage of hypertension, increased basal aldosterone levels (> 20 ng/dl), aldosterone not inhibited after saline treatment (> 10 ng/dl), and a diagnosis of PA.

**Table 3:** Positional test

	Renin (ng/l)	Angiotensin II (ng/l)	Aldosterone (ng/dl)	ARR
2017 Supine position	6.86 (4–24)	107.96 (25–129)	11.40 (10–160)	1.66
Upright position	10.97 (4–38)	119.46 (49–252)	10.16 (40–310)	0.97
2020 Supine position	23.99 (4–24)	99.95 (25–129)	20.65 (10–160)	0.86
Upright position	44.78 (4–38)	118.42 (49–252)	25.51 (40–310)	0.57

**Table 4:** the inhibitory test with saline

	Renin (ng/l)	Angiotensin II (ng/l)	Aldosterone (ng/dl)	ARR
Before infusion	23.99	99.95	20.65	0.86
After infusion	26.92	101.04	23.72	0.88

**Fig. 2:**
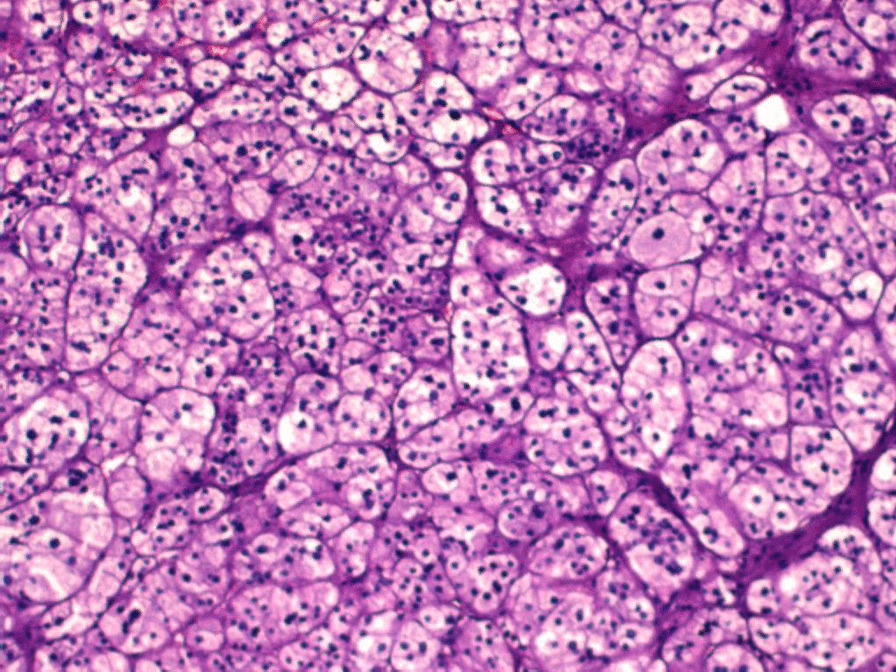
Pathological picture of left adrenal adenoma

For the bilateral adrenal multiple adenomas, we further performed bilateral adrenal venous sampling (AVS). For hormonal examination, blood specimens were collected from 4 different sites, including the left adrenal vein, right adrenal vein, postcava and precava. The test results are shown in Table 5. The selectivity index of right and left adrenal venous cannulation was 2.9 and 2.22 (Table 5), respectively, and judged as successful [2]. When we compared the ratio of aldosterone-over-cortisol (A/C) obtained in both adrenal veins, the lateralization index was 1.01 (< 2) (Table 5), indicating no dominant secretion [8]. Because AVS showed no dominant secretion of either adrenal gland, it was recommended that the patient take oral medication to control hypertension. We considered that bilateral adrenal adenomas were functional, and the patient had poor blood pressure control with a variety of antihypertensive drugs in the past, serious target organ damage, and hypokalaemia that was difficult to correct. On the basis of the evidence, after obtaining the consent of the patient, the patient was transferred to the urology department for laparoscopic resection of the left large adrenal adenoma. Postoperative pathology was indicative of adrenal adenoma (Fig. 2). Postoperative blood potassium was 4.12 mmol/l. After the operation, the blood pressure of the patient was more easily controlled than before. After 6 months, blood pressure was controlled at 130/90 mmHg with three kinds of antihypertensive drugs (nifedipine controlled-release tablets, arotinolol, spironolactone).

**Table 5:** Adrenal vein blood sampling

	Left adrenal vein	Right adrenal vein	postcava	precava
Cortisol (nmol/l) Average value	568.93 570.82 (569.875)	777.69 709.76 (743.725)	259.25 253.41 (256.33)	333.76 351.85 (342.805)
Aldosterone (pg/ml) Average value	1639.5 1971.5 (1805.5)	2446.5 2326.00 (2386.25)	208.4 168 (188.2)	170.5 166.4 (168.45)
A/C	3.17	3.21	0.73	0.49
SI	2.22	2.9		

A, aldosterone; A/C, a ratio of aldosterone-over-cortisol; C, cortisol; SI, selectivity index

## Discussion and conclusions

We reported a case of primary aldosteronism. Although the initial screening test (ARR) was negative, we further confirmed the diagnosis with the inhibitory test with saline and concluded that PA could not be excluded when ARR was negative. According to the guidelines [6], PA can be diagnosed by a positive orthostatic ARR and confirmatory tests, but many factors affect the results of PA screening and confirmatory tests. As a clinical disease, the diagnosis of PA should be based on complete clinical manifestations, laboratory examination, AVS, histopathology and treatment response.

Aldosterone is a hormone regulating blood volume in the human body which regulated by renin-angiotensin system (RAS), angiotensin II, adrenocorticotropic hormone(ACTH) and potassium ions. In PA, the relative high blood volume caused by increased aldosterone further inhibits RAS; aldosterone secretion is relatively independent of the inhibited RAS, but can be affected by body position, hormone [9] and various receptor ligands [10], which may lead to great variability of aldosterone levels in PA patients. It has been reported that chronic stress induced by ACTH leads to ACTH dependent hyperaldosterone secretion in hypertensive patients without PA [11]. When hypokalemia and resistant hypertension exist, the possibility of PA is very high. In the case of low renin level, the production of aldosterone at any level suggests that there may be potential PA. Two studies have shown that even normal aldosterone levels(< 10 ng/dl) may be an indicator of PA [12, 13]. Oelkers et al. [14] reported three patients with PA complicated by renal insufficiency whose plasma renin was not inhibited. In these patients, plasma renin activity was in the normal/high normal range, which may have been related to renal vascular injury leading to glomerular ischaemia and renin escaping excess aldosterone inhibition. In the diagnosis of PA with the ARR, the effect of renal damage on plasma renin concentration (PRC) may cause an increase in the PRC, which leads to a false lower ARR value, thus masking PA [15].

This patient had mild renal insufficiency, which may have been a cause of the negative ARR. There are different reports on the ARR cut-off point in the diagnosis of PA in domestic and foreign studies [4]. At present, the ARR cut-off point recommended by previous studies is used in the screening of PA in patients with hypertension, and whether this cut-off point is in line with the local population has not been further discussed; therefore, PA may be missed in some people with an ARR lower than the cut-off point. In 2017, Zorzl et al [16] reported a 27-year-old patient with hypertension who was diagnosed with essential hypertension after a negative ARR was determined. Many years later, the patient repeatedly went to the hospital due to poor blood pressure control (taking four kinds of antihypertensive drugs at the same time) and hypokalaemia. After 14 years, the ARR was positive, and PA was confirmed by the inhibitory test with saline and AVS. After the operation, blood pressure and potassium levels returned to normal. Therefore, when the ARR is negative, PA cannot be easily excluded. Comprehensive analysis and diagnosis should be based on the medication and clinical conditions of patients.

Bilateral adrenal diseases can be divided into three types: idiopathic adrenal hyperplasia (IAH), bilateral aldosterone-producing adenoma (APA) and glucocorticoid-remediable aldosteronism (GRA) [6]. It is difficult to distinguish bilateral APA from IAH even with successful AVS [17]. In 2016, Japanese scholars used segmental AVS (S-AVS) to confirm that the veins in the tumour segment secreted too much aldosterone and inhibited the bilateral secretion of veins in the nontumour segment, leading to the diagnosis of bilateral APA [17]. In theory, bilateral APA can also be cured by surgery, but there are few successful cases reported in the literature. Few patients with bilateral APA underwent surgery and were cured by surgery [17–20].

Guidelines recommend mineralocorticoid receptor (MR) antagonists for PA patients with bilateral adrenal disease [6]. However, it has been reported that the cardiovascular risk of PA patients is higher than that of patients with essential hypertension, despite good blood pressure control [21]. When MR antagonists are used, the decline in glomerular filtration rate increases the risk of hyperkalaemia, and large doses of MR antagonists, which can block MR sufficiently, are also limited [22]. The use of high-dose spironolactone also has anti-androgen side effects. A Japanese national survey also showed that surgical treatment can improve hypertension and hypokalaemia in unilateral and bilateral adrenal lesions [23]. Scholars have raised the following question: Can adrenalectomy reduce aldosterone exposure in PA patients with bilateral adrenal diseases? [24] There is a lack of effective clinical research to answer this question, and more prospective studies are needed. For PA patients whose blood pressure cannot be controlled sufficiently or whose hypokalaemia is difficult to correct or whose renal function is impaired and the dosage of MR antagonist cannot be increased to the maximum, scholars propose the choice of unilateral adrenalectomy to reduce the severity of the disease, coupled with long-term MR antagonist treatment [24]. We performed a unilateral adenoma resection for this patient, who continued taking spironolactone to control blood pressure. Currently, blood pressure is well controlled, and good clinical effects have been achieved.

Finally, we summarize some experience gained from this case and some hints for future clinical work. Clinically, for patients with atypical suspected PA, we should not rely only on hormone level and ARR screening to exclude PA, but we should make a judgement after comprehensive evaluation of clinical manifestations and laboratory and imaging examinations. For PA patients, long-term clinical follow-up observation, including the monitoring of drug side effects, blood pressure and various cardiovascular complications, is also needed, regardless of the choice of drugs or surgical treatment.

## Data Availability

All relevant data supporting the conclusions of this article are included within the article.

## Abbreviations

PA: Primary aldosteronism
ARR: Aldosterone-to-renin ratio
CT: Computed tomography
AVS: Adrenal venous sampling
S-AVS: Segmental AVS
PRC: Plasma renin concentration
IAH: Idiopathic adrenal hyperplasia
APA: Aldosterone-producing adenoma
GRA: Glucocorticoid-remediable aldosteronism
MR: Mineralocorticoid receptor
A: Aldosterone
C: Cortisol
A/C: A ratio of aldosterone-over-cortisol
SI: Selectivity index
